# Mental Health and Wellbeing of Retired Elite and Amateur Rugby Players and Non-contact Athletes and Associations with Sports-Related Concussion: The UK Rugby Health Project

**DOI:** 10.1007/s40279-021-01594-8

**Published:** 2021-11-18

**Authors:** Karen Hind, Natalie Konerth, Ian Entwistle, Patria Hume, Alice Theadom, Gwyn Lewis, Doug King, Thomas Goodbourn, Marianna Bottiglieri, Paula Ferraces-Riegas, Amanda Ellison, Paul Chazot

**Affiliations:** 1 Department of Sport and Exercise Sciences, Durham University, 42 Old Elvet, Durham, DH1 3HN UK; 2 Wolfson Research Institute for Health and Wellbeing, Durham University, Durham, UK; 3 Sports Performance Research Institute New Zealand (SPRINZ), Faculty of Health and Environmental Science, Auckland University of Technology, Auckland, New Zealand; 4 TBI Network, Auckland University of Technology, Auckland, New Zealand; 5 National Institute for Stroke and Applied Neuroscience, Auckland University of Technology, Auckland, New Zealand; 6 Health and Rehabilitation Research Institute, Faculty of Health and Environmental Science, Auckland University of Technology, Auckland, New Zealand; 7 School of Science and Technology, University of New England, Armidale, NSW Australia; 8 Department of Biosciences, Durham University, Stockton Road, Durham, DH1 3LE UK; 9 Department of Psychology, Durham University, Durham, UK

## Abstract

**Background:**

Concerns have intensified over the health and wellbeing of rugby union and league players, and, in particular, about the longer-term effects of concussion. The purpose of this study was to investigate whether there were differences in mental health, sleep and alcohol use between retired elite and amateur rugby code players and non-contact athletes, and to explore associations with sports-related concussion.

**Methods:**

189 retired elite (ER, *n* = 83) and amateur (AR, *n* = 106) rugby code players (rugby union *n* = 145; rugby league *n* = 44) and 65 former non-contact athletes (NC) were recruited to the UK Rugby Health Project between 2016 and 2018. Details on sports participation and concussion history were obtained by questionnaire, which also included questions on mental health, anger, sleep, mood, alcohol use, social connections and retirement from injury. Data were compared between sports groups (ER, AR and NC), between exposure of three or more or five or more concussions and for years in sport.

**Results:**

ER reported more concussions than AR (5.9 ± 6.3 vs. 3.7 ± 6.3, *p* = 0.022) and NC (0.4 ± 1.0, *p* < 0.001). ER had a higher overall negative mental health score (indicating poor mental health) than AR (10.4 ± 6.3 vs. 7.4 ± 6.5, *d* = 0.47, *p* = 0.003) and NC (7.1 ± 4.8, *d* = 0.57, *p* = 0.006) and a lower overall positive score (indicating good mental health) than NC (8.9 ± 4.1 vs. 10.7 ± 3.4, *d* = 0.46, *p* = 0.021). Negative scores were highest and positive scores lowest in those reporting three or more concussions (*d* = 0.36, *p* = 0.008; *d* = 0.28, *p* = 0.040, respectively) or five or more concussions (*d* = 0.56, *p* < 0.001; *d* = 0.325, *p* = 0.035, respectively). Reported symptoms for sleep disruption were more prevalent in ER than NC, and in former athletes with three or more concussions (*d* = 0.41–0.605, *p* < 0.05). There were no significant differences in alcohol score (*p* = 0.733). Global anger score and covert anger expression was higher in former athletes with five or more concussions (*d* = 0.32, *p* = 0.035; *d* = 0.37, *p* = 0.016). AR reported greater attachment to friends than NC (*d* = 0.46, *p* = 0.033) and 20% of ER reported that they would not turn to anyone if they had a problem or felt upset about anything.

**Conclusion:**

There was a significantly higher prevalence of adverse mental health and sleep disruption in ER and in former athletes who reported a higher number of concussions. Anger and irritability were more prevalent in former athletes with a history of five or more concussions. Strategies are needed to address mental health and sleep disturbance in elite rugby code athletes, who are also less likely to seek help should they need it. Further research is needed to elucidate causation, and the neurobiological connection between concussion, sub-concussions and longer-term psychological health and wellbeing.

**Supplementary Information:**

The online version contains supplementary material available at 10.1007/s40279-021-01594-8.

## Key Points

**Table:** 

Retired elite rugby code players scored consistently worse for psychological signs of depression, anxiety and irritability when compared to amateur rugby code and non-contact athlete groups. Retired elite players also reported a higher number of sports-related concussions.
Sleep disruption was more prevalent in retired elite rugby code players compared to retired amateur rugby code players and non-contact athletes.
Former athletes reporting a history of three or more concussions scored significantly worse for psychological signs of depression and anxiety and for sleep disruption. Those with a history of five or more concussions had significantly higher global anger scores, higher covert anger expression scores, and almost double the prevalence of depression, anxiety and irritability.
One in five elite rugby code athletes reported that they would not seek help from anyone if they had a problem or were upset.

## Background

Rugby is an intermittent contact team-sport involving numerous collisions and tackles. It is one of the world’s most popular team sports [1], with over 9.6 million players (including 2.7 million female) across 123 countries [2]. Two codes of rugby exist, rugby league and rugby union. The rugby codes have different rules concerning the number of players on the field and details of breakdown and set-piece play. Rugby union is the more popular of the two codes, with 2.5 million players participating in the UK and Ireland alone [2]. Rugby league has seen growth for three consecutive years in the UK, following Sport England’s denotation as a ‘priority sport’ [3]. Rugby participation in both codes is at an all-time high, but concerns over player safety, wellbeing and health have intensified due to an increasing injury rate at both the amateur and the professional levels of participation [4–6].

Several studies have identified that, particularly at elite levels, athletes are vulnerable to physical, psychological, interpersonal and structural stressors that could undermine their mental health [7–9]. In current professional male rugby players (rugby union, rugby league and rugby sevens), 11% reported feelings of distress, 12% reported sleep disruption, 22% reported adverse alcohol use, and 28% reported feeling anxious and/or depressed [10]. In both rugby union and rugby league, players who sustained a severe musculoskeletal injury were twice as likely to develop anxiety and depression symptoms over 12 months, compared to players who had not sustained severe injuries [11]. In particular, the risk of developing adverse mental health symptoms during the season has been reported as 1.5 times greater in professional rugby union players who sustain a concussion (mild traumatic brain injury) when compared with those who do not [11]. Similarly, in active professional rugby league players, sustaining three or more sport-related concussions has been associated with a twofold greater risk for developing adverse mental health symptoms [12]. There is also concern about the cumulative effects of sub-concussive head impacts over an athlete's career [13].

Although there are a growing number of studies on mental health in current rugby code players, few studies have investigated the mental health of retired rugby union players and none have explored the mental health of retired rugby league players. Retired professional rugby union players from France, Ireland and South Africa have shown a higher prevalence of mental health disorders when compared with the general population, particularly for distress, anxiety and/or depression symptoms, which were reported by 25–28% of former players [14]. In contrast, another study reported no differences in measures of mental health and stress between retired rugby union players and a non-rugby group [15].

Few studies have explored associations between mental health and concussion history in contact-sport athletes. One study in retired elite rugby union players reported increased depression scores with a higher number of reported concussions [16], and in retired American Football players, the risk of depression was greater in former players with a history of three or more concussions [17]. In addition to the lack of research on the long-term risks of concussion on the mental health of retired rugby code players, no published study to date has examined relationships with anger, sleep and alcohol use, or has explored these outcomes in both codes of rugby and at both the elite and the amateur levels of participation. Therefore, the purpose of this study was to investigate mental health, sleep and alcohol use, and associations with concussion, in retired UK elite and amateur rugby code athletes and to compare these to retired, age-matched, non-contact athletes.

## Methodology

### Study Design and Setting

The research was a cross-sectional analysis of 254 participants from the UK Rugby Health Project. With a multidisciplinary research focus, the UK Rugby Health Project was initiated in 2016 as an extension to the inaugural New Zealand (NZ) Rugby Health Project [18] and in response to calls for international efforts to acquire further knowledge and understanding of the health and wellbeing of contact-sport athletes. To date, published findings on this cohort include cumulative injuries and physical health in retirement [19] and neurovascular alterations associated with concussion [20]. The UK Rugby Health project was approved by Durham University and Leeds Beckett University Research Ethics Committees, and performed in accordance with the Standards of Ethics outlined in the Declaration of Helsinki. Informed consent was obtained from all participants in the study, and to protect the identity of participants, all data were obtained anonymously through online questionnaires.

### Study Participants

Retired male rugby code players and non-contact-sport athletes took part in the study and were recruited from September 2016 to December 2018 using past player/athlete associations, printed and televised media reports, word of mouth and social media. The sample size calculations were computed according to published findings of depression and anxiety scores in retired mixed contact and non-contact athletes [21], and based on a power of 80%, confidence interval of 95%, and precision of 5% to detect differences between the groups. For scores of depression, at least 19 participants were required for each group, and for scores of anxiety, at least 29 participants were required for each group. The final study sample comprised of 83 retired elite rugby code (ER) players (49 elite rugby union; 34 elite rugby league), 106 retired amateur rugby code (AR) players (96 amateur rugby union; 10 amateur rugby league) and 65 retired non-contact (NC) athletes. ER and AR participants were recruited first to facilitate age-matching of NC. ER players had competed at international or national level, and/or at professional or semi-professional level. AR players had only played at club or regional level and had not received payment for playing. Retired elite players had stopped playing any level of contact rugby. The retired amateur rugby group only included those who had previously played at amateur level, and did not include former elite players. The NC group had only participated in non-contact sport at amateur or elite level. They may have participated in contact sport during school (to age 16 years) but were excluded if they had taken part in any contact sport post-school. Over half of the retired NC athletes reported cricket as their main sport (*n* = 35) and the remainder reported running, swimming or cycling (*n* = 30).

### Procedures

Information on engagement in sport, demographic information, concussion injury and current mental health and wellbeing status were obtained from a general health e-questionnaire (see Electronic Supplementary Material (ESM)) that was adapted from the NZ RugbyHealth study of 366 former athletes [18]. Adaptations were made to the questionnaire to reflect competition structure in the UK, and to include an additional question on concussions sustained outside of sport. The questionnaire has six sections (Sect. 1: Demographics; Sect. 2: Sport Participation; Sect. 3: Ability to Perform Tasks; Sect. 4: Injury History; Sect. 5: Health, Lifestyle and Wellbeing; Sect. 6: Other Details e.g., education, relationship status) with a total of 97 questions taking approximately 40 min to complete. The questionnaire was available online from September 2016 to December 2018 and could also be accessed as a paper version, although this was only utilized by one participant and responses were entered manually into the study database.

Sport-related concussion was defined as “arising from a direct knock to the head or indirect (such as arising from whiplash)”. Participants were also asked to report on non-sports-related concussions, and these were considered in the analysis. The questions concerning mental health and wellbeing were included in Sect. 5 of the questionnaire and were adapted from a series of validated tools as follows. Questions concerning alcohol were derived from the Alcohol Use Disorders Identification Test (AUDIT-C) [21]. AUDIT-C has been well studied, with results suggesting good levels of reliability (Cronbach *α* > 0.80) [22]. Three questions concerning sleep quality were derived from the Insomnia Severity Index [23–25]. Participants were asked to report if, over the past 4 weeks, they had experienced difficulties falling asleep, waking up too early, or waking up in the night and taking a long time to get back to sleep. Answers were given on a 5-point Likert scale and were coded as follows: 0—None, 1—Rarely, 2—Sometimes, 3—Often, 4—Always. Negative and positive feelings were assessed using questions from the General Health Questionnaire (GHQ-12) and the Affectometer-2. The GHQ-12 is a screening tool for identifying psychological disorders in the general population. It assesses how a person is feeling and any impact on their ability to carry out normal functions as a result. Participants rate each feeling/experience over the past month on a 4-point Likert scale of 0 (never) to 3 (always) [26, 27]. The Affectometer-2 assesses positive mental health and feelings of happiness [26, 28]. Responses were made on a 4-point Likert scale: 0—Never, 1—Sometimes, 2—Often, 3—Always. Questions relating to irritability and anger were derived from the Spielberger Anger Expression Scale [29]. The questionnaire includes two subscales, Anger/In and Anger/Out, measuring anger suppression and overt anger expression, respectively. Both subscales have been consistently verified [30–32]. Answers were reported on a 4-point Likert scale: 0—Never, 1—Sometimes, 2—Often, 3—Always. Participants were also asked if they had formerly or currently suffered from anxiety, depression, or irritability. As a measure of community integration and social support, participants were asked about their involvement in activities with family, friends and former sports clubs and were asked to identify whom, if anyone, they would turn to if they had a problem or were upset.

### Statistical Analyses

For the primary analysis, data were compared between retired ER, AR and NC groups. Data are presented as the mean ± standard deviation. Although most of the data were ordinal in nature and some variables were skewed, it has been shown that, given sufficient sample sizes, parametric approaches are sufficiently robust to withstand these violations of assumptions [33, 34]. Furthermore, the use of exact values rather than ranks provides the parametric approach with more power. Therefore, one-way analysis of variance (ANOVA) was utilised with post hoc pairwise comparisons performed via Tukey’s honestly significant difference test when significant differences were found. Effect size was calculated as partial eta squared for the ANOVA tests and Cohen's d for the pairwise comparisons. The thresholds for the partial eta squared values are 0.01 for a small effect, 0.06 for a medium effect, and 0.14 for a large effect. For Cohen’s *d*, the thresholds assumed are 0.2 for a small effect, 0.5 for a medium effect, and 0.8 for a large effect. When responses were binary in nature, the chi-square test of independence was performed, with Fisher’s exact test utilised when expected cell counts were less than 5. Data were compared between individuals who did or did not retire from their sport due to injury. In addition, data were compared between those who reported exposure of less than three (< 3) and three or more (≥ 3) or less than five (< 5) and five or more (≥ 5) sport-related concussions. These cut-offs represented the 50th and 75th percentiles for sports-related concussions in the current study. Data were also compared between rugby players for years of exposure to head impacts using the variable of 'years in sport', with cut-offs of 25 years and 30 years, representing the 50th and 75th percentiles in the current study, respectively. Comparisons were made using independent-samples *t* tests and the chi-square test of independence. Data were analysed using SPSS for Windows (IBM SPSS, Version 26, Armonk, NY, USA) and Microsoft Excel (Microsoft Corporation 2016, Version 1902, Redmond, WA, USA). Statistical significance was identified at *p* < 0.05.

## Results

### Demographic Characteristics

Participant demographics are given in Table 1. ER and AR retired at a younger age compared to NC (*p* < 0.001; *p* = 0.004 respectively). Injury was the primary cause of retirement in 56% ER, 52% AR and 29% NC athletes (ER > NC *p* = 0.001, AR > NC *p* = 0.005) and 9% of rugby code players retired due to concussion. Further details on injuries leading to retirement are published elsewhere [19].

**Table 1:** Participant demographics of retired elite rugby codes, amateur rugby codes, non-contact, and combined sports participants

	Age, y		Starting age, y	Retirement age, y	Years in sport
	Mean ± SD	Range	Mean ± SD	Mean ± SD	Mean ± SD
Elite rugby code players (*n* = 83)	43.4 ± 9.4*^	21.5–73.5	8.8 ± 2.9*^	33.2 ± 5.6^	24.3 ± 6.5^
Amateur rugby code players (*n* = 106)	48.3 ± 11.0	24.2–82.2	10.6 ± 3.6	36.4 ± 9.6^#^	25.7 ± 9.3^#^
Non-contact athletes (*n* = 65)	48.7 ± 12.9	24.3–72.9	10.7 ± 4.8	41.9 ± 12.7	32.0 ± 13.4
Combined (*n* = 254)	46.8 ± 11.3	21.5–82.2	10.0 ± 3.8	36.3 ± 9.6	26.4 ± 9.7

Significant difference (*p* < 0.05) between *Elite Rugby (ER) vs. Amateur Rugby (AR), ^ER vs. Non-Contact (NC), ^#^AR vs. NC

### Mental Health Conditions

The percentage of individuals who reported being either previously or currently suffering from irritability, depression or anxiety varied across ER, AR and NC (see Table 2). A greater proportion of ER reported previous or current irritability, depression and anxiety, with approximately half of these individuals reporting suffering from irritability and depression.

**Table 2:** Prevalence of reported mental health conditions for participants in elite rugby codes, amateur rugby codes and non-contact-sporting codes

	Elite rugby codes (%)	Amateur rugby codes (%)	Non-contact athletes (%)	Difference
Irritability	52	43	18	ER vs. NC *p* < 0.001 AR vs. NC *p* = 0.002
Depression	49	34	21	ER vs. NC *p* = 0.001 ER vs. AR *p* = 0.043
Anxiety	42	23	31	ER vs. AR *p* = 0.009

*ER* elite rugby, *AR* amateur rugby, *NC* non-contact

### Psychological Signs of Depression and Anxiety

There were significant differences between groups for overall negative and positive feeling scores, and in a range of individual positive and negative feelings items (see Table 3). These outcomes related to feelings experienced in the last month. ER had a higher overall negative feelings score than AR (*d* = 0.464, *p* = 0.003) and NC (*d* = 0.569, *p* = 0.006) and a lower overall positive feelings score compared to NC (*d* = 0.463, *p* = 0.021). The percentage of individuals who reported often or always feeling satisfied (ER: 46%, AR: 61%, NC: 70%), miserable (ER: 22%, AR: 11%, NC: 7%), depressed (ER: 16%, AR 8%, NC 7%), down (ER: 20%, AR: 8%, NC: 4%), ‘life is hardly worth living’ (ER: 10%, AR: 4%, NC: 0%), content (ER: 58%, AR: 72%, NC: 70%), lost sleep over worry (ER: 29%, AR: 17%, NC: 12%), and unable to concentrate (ER: 23%, AR: 14%, NC: 7%) varied between groups, with psychological signs of anxiety and/or depression consistently higher in ER.

**Table 3:** Psychological signs of depression and anxiety for participants in elite rugby codes, amateur rugby codes and non-contact-sporting codes

	Elite rugby codes (mean ± SD)	Amateur rugby codes (mean ± SD)	Non-contact athletes (mean ± SD)	Difference (*p* value)	Effect size (partial Eta squared)	Pairwise effect size (Cohen’s *d*)
Everything is going right for me	1.2 ± 0.8	1.4 ± 0.8	1.4 ± 0.6	*p* = 0.115	*η*^2^ = 0.018	
My life is on the right track	1.5 ± 0.9	1.6 ± 0.8	1.8 ± 0.8	*p* = 0.081	*η*^2^ = 0.021	
Confident	1.6 ± 0.9	1.8 ± 0.8	1.9 ± 0.7	*p* = 0.091	*η*^2^ = 0.020	
Happy	1.7 ± 0.8	1.8 ± 0.6	1.9 ± 0.6	*p* = 0.205	*η*^2^ = 0.013	
Satisfied	1.4 ± 0.8	1.7 ± 0.7	1.8 ± 0.7	ER < NC *p* = 0.011	*η*^2^ = 0.038	ER < NC *d* = 0.484
Content	1.6 ± 0.8	1.9 ± 0.8	1.9 ± 0.8	ER < AR *p* = 0.048; ER < NC * p* = 0.048	*η*^2^ = 0.031	ER < AR *d* = 0.345; ER < NC *d* = 0.419
I’ve made a mess of things again	0.8 ± 0.8	0.5 ± 0.6	0.6 ± 0.6	*p* = 0.052	*η*^2^ = 0.025	
Nothing is much fun anymore	0.8 ± 0.8	0.6 ± 0.8	0.5 ± 0.6	*p* = 0.139	*η*^2^ = 0.016	
Nothing goes right with me	0.6 ± 0.8	0.4 ± 0.7	0.4 ± 0.6	*p* = 0.066	*η*^2^ = 0.023	
Unable to make decisions	0.8 ± 0.7	0.6 ± 0.7	0.5 ± 0.7	*p* = 0.057	*η*^2^ = 0.024	
Unable to concentrate	1.0 ± 0.7	0.7 ± 0.7	0.7 ± 0.7	ER > AR *p* = 0.020; ER > NC * p* = 0.015	*η*^2^ = 0.041	ER > AR *d* = 0.396; ER > NC * d* = 0.491
Life is hardly worth living	0.4 ± 0.7	0.2 ± 0.5	0.1 ± 0.3	ER > AR *p* = 0.024; ER > NC * p* = 0.003	*η*^2^ = 0.050	ER > AR *d* = 0.356; ER > NC *d* = 0.559
Lost sleep over worry	1.1 ± 0.8	0.7 ± 0.8	0.6 ± 0.7	ER > AR *p* < 0.001; ER > NC * p* = 0.001	*η*^2^ = 0.076	ER > AR *d* = 0.566; ER > NC *d* = 0.652
Miserable	1.0 ± 0.7	0.8 ± 0.7	0.6 ± 0.6	ER > NC *p* = 0.011	*η*^2^ = 0.039	ER > NC *d* = 0.496
Depressed	0.8 ± 0.8	0.5 ± 0.8	0.4 ± 0.6	ER > AR *p* = 0.009; ER > NC * p* = 0.023	*η*^2^ = 0.043	ER > AR *d* = 0.424; ER > NC *d* = 0.496
Down	1.1 ± 0.7	0.7 ± 0.6	0.7 ± 0.5	ER > AR *p* = 0.002; ER > NC * p* = 0.008	*η*^2^ = 0.058	ER > AR *d* = 0.495; ER > NC *d* = 0.522
Upset	0.9 ± 0.6	0.8 ± 0.7	0.7 ± 0.5	*p* = 0.130	*η*^2^ = 0.017	
Pressured	1.2 ± 0.7	1.0 ± 0.8	1.0 ± 0.7	*p* = 0.128	*η*^2^ = 0.017	
Overall negative feelings score	10.4 ± 6.3	7.4 ± 6.5	7.1 ± 4.8	ER > AR *p* = 0.003; ER > NC * p* = 0.006	*η*^2^ = 0.055	ER > AR *d* = 0.464; ER > NC *d* = 0.569
Overall positive feelings score	8.9 ± 4.1	10.2 ± 3.9	10.7 ± 3.4	ER < NC *p* = 0.021	*η*^2^ = 0.033	ER < NC *d* = 0.463

Overall scores were calculated as a sum of the respective scores for positive and negative feelings

*ER* elite rugby, *AR* amateur rugby, *NC* non-contact, *SD* standard deviation

### Sleep

The percentage of individuals who reported having sleep difficulties often/always was higher in ER compared to AR and NC for ‘difficulty falling asleep’ (ER: 17%, AR: 6%, NC: 7%), ‘waking in the night and taking a long time to fall back to sleep’ (ER: 34%, AR: 19%, NC 17%) and ‘waking up too early’ (ER: 35%, AR 19%, NC 12%) (see Table 4).

**Table 4:** Sleep-related responses for participants in elite rugby codes, amateur rugby codes and non-contact-sporting codes

	Elite rugby codes (mean ± SD)	Amateur rugby codes (mean ± SD)	Non-contact athletes (mean ± SD)	Difference	Effect size (partial Eta squared)	Pairwise effect size (Cohen’s *d*)
Difficulty falling asleep	1.2 ± 1.3	1.0 ± 1.0	0.9 ± 1.0	*p* = 0.174	*η*^2^ = 0.015	
Waking in the night and taking a long time to get back to sleep	1.7 ± 1.2	1.4 ± 1.1	1.0 ± 1.2	ER > NC *p* = 0.001	*η*^2^ = 0.053	ER > NC *d* = 0.605
Waking up too early	1.8 ± 1.2	1.6 ± 1.0	1.3 ± 1.1	ER > NC *p* = 0.032	*η*^2^ = 0.026	ER > NC *d* = 0.414

*SD* standard deviation

### Alcohol

There was no difference in the AUDIT-C score between groups (see Table 5). ER reported drinking less frequently than NC (*d* = 0.464, *p* = 0.022). Overall, AUDIT-C scores for higher risk from alcohol consumption (≥ 5) were prevalent in 59% of ER, 64% of AR, and 53% of NC (*p* = 0.381).

**Table 5:** Alcohol consumption and AUDIT-C score for participants in elite rugby codes, amateur rugby codes and non-contact-sporting codes

	Elite rugby codes (mean ± SD)	Amateur rugby codes (mean ± SD)	Non-contact athletes (mean ± SD)	Difference	Effect size (partial Eta squared)	Pairwise effect size (Cohen’s *d*)
How often do you drink?	2.3 ± 1.1	2.6 ± 1.1	2.8 ± 1.1	ER < NC *p* = 0.022	*η*^2^ = 0.033	ER < NC *d* = 0.464
How many drinks containing alcohol do you have on a typical day when drinking?*	1.4 ± 1.2	1.2 ± 1.0	1.0 ± 1.0	*p* = 0.062	*η*^2^ = 0.029	
How often do you have six or more drinks on one occasion?	1.6 ± 0.9	1.5 ± 1.0	1.3 ± 1.0	*p* = 0.230	*η*^2^ = 0.012	
AUDIT-C score	5.3 ± 2.4	5.3 ± 2.2	5.0 ± 2.4	*p* = 0.733	*η*^2^ = 0.022	

Questions and scoring based on the Alcohol Use Disorders Identification Test (AUDIT)

*ER* elite rugby, *AR* amateur rugby, *NC* non-contact, *SD* standard deviation

*0–2 = 0, 3–4 = 1, 5–6 = 2, 7–9 = 3, 10 +  = 4'

### Irritability and Anger

There were no differences in global anger scores and in both overt and covert expression of anger across groups (see Table 6).

**Table 6:** Irritability and anger for participants in elite rugby codes, amateur rugby codes and non-contact-sporting codes

	Elite rugby codes (mean ± SD)	Amateur rugby codes (mean ± SD)	Non-contact athletes (mean ± SD)	Difference	Effect size (partial Eta squared)
Anger/Out score	6.5 ± 3.8	5.8 ± 3.5	5.5 ± 3.3	*p* = 0.254	*η*^2^ = 0.011
Anger/In score	7.4 ± 4.3	6.5 ± 3.6	7.1 ± 3.6	*p* = 0.255	*η*^2^ = 0.011
Overall anger score	13.8 ± 6.7	12.3 ± 5.6	12.6 ± 4.6	*p* = 0.172	*η*^2^ = 0.015

*SD* standard deviation

### Social Connection and Sources of Support

AR reported more involvement with their friends than NC (*d* = 0.445, *p* = 0.033) (see Table 7). The proportion of individuals who responded that they would try to talk to someone if they had a problem that was bothering them (vs. “would try not to talk about it”) was not significantly different across ER (45%), AR (54%) and NC (56%) groups (*p* = 0.338). However, the percentage of participants who stated that they “would not turn to anyone if they had a problem or felt upset about something” were significantly greater in ER (20%) when compared to AR (8.8%; *p* = 0.030) and NC (6.8%; *p* = 0.028).

**Table 7:** Social connection in elite rugby codes, amateur rugby codes and non-contact-sporting codes

	Elite rugby codes (mean ± SD)	Amateur rugby codes (mean ± SD)	Non-contact athletes (mean ± SD)	Difference	Effect size (partial Eta squared)	Pairwise effect size (Cohen’s *d*)
Involvement with family	4.1 ± 1.2	4.2 ± 1.1	4.0 ± 1.1	*p* = 0.482	*η*^2^ = 0.006	
Involvement with friends	3.5 ± 1.4	3.8 ± 1.2	3.3 ± 1.0	AR > NC *p* = 0.033	*η*^2^ = 0.027	AR > NC *d* = 0.445
Involvement with work/university	3.3 ± 1.5	3.4 ± 1.6	3.1 ± 1.6	*p* = 0.614	*η*^2^ = 0.003	
Involvement with former sports friends/clubs	2.0 ± 1.6	2.3 ± 1.7	2.1 ± 1.6	*p* = 0.316	*η*^2^ = 0.010	

*SD* standard deviation

### Retirement Due to Injury

Current or previous depression and anxiety were more prevalent in former athletes who reported they retired because of injury when compared to those who did not (46% vs. 30%, *p* = 0.017; 38% vs. 25%, *p* = 0.045, respectively). There were no differences observed in the reported prevalence of irritability or overall anger subscales between athletes who reported they retired because of injury and those who did not (47% vs. 35%, *p* = 0.074; 13.3% vs. 12.6%, *p* = 0.365).

### Concussion and Years in Sport

The number of sports-related concussions was greater in ER (5.9 ± 6.3) and AR (3.7 ± 6.3) compared to NC (0.4 ± 1.0) (both *p* ≤ 0.001), and greater in ER compared to AR (*p* = 0.022). There were no significant differences observed in the number of sports-related concussions reported by ER (*p* = 0.085) and AR (*p* = 0.462) rugby league players when compared with rugby union players. There were no differences observed in the number of concussions sustained outside of sport between retired athlete groups (ER: 19%, AR: 19%, NC: 19%; *p* = 0.997).

When data were analysed by concussion history rather than athlete group, reported irritability, depression and anxiety, overall negative feelings score, and symptoms of sleep disruption were greater in those who had ≥ 3 (50th percentile) (*d* = 0.356) or ≥ 5 (75th percentile) (*d* = 0.556) sport-related concussions, while scores for positive feelings were lower (*d* = 0.275 and 0.321, respectively, all *p* < 0.05; see Table 8 and ESM). Differences between concussion groups were significantly more pronounced when the cut-off of five sports-related concussions was utilised instead of three, particularly for anger subscales (see Table 8 and ESM). There were no differences in AUDIT-C score and no differences in social connection between concussion groups (see ESM).

**Table 8:** Mental health and wellbeing scores for retired athletes with or without ≥ 3 or ≥ 5 sports-related concussions (mean ± SD or percentage)

	At least 3 sport-related concussions (*n* = 99)		Difference	Effect Size (Cohen’s *d*)	At least 5 sport-related concussions (*n* = 60)			Difference	Effect size (Cohen’s *d*)
	Yes	No			Yes	No			
Irritability	54%	30%	*p* = 0.001*		63%		33%	*p* < 0.001*	
Depression	47%	30%	*p* = 0.010*		55%		30%	*p* = 0.001*	
Anxiety	37%	27%	*p* = 0.116		44%		26%	*p* = 0.012*	
Difficulty falling asleep	1.2 ± 1.2	1.0 ± 1.0	*p* = 0.157	*d* = 0.200	1.3 ± 1.3		1.0 ± 1.0	*p* = 0.133	*d* = 0.270
Waking in the night and taking a long time to get back to sleep	1.8 ± 1.1	1.2 ± 1.2	*p* < 0.001*	*d* = 0.523	1.8 ± 1.2		1.4 ± 1.2	*p* = 0.030*	*d* = 0.329
Waking up too early	1.9 ± 1.1	1.5 ± 1.1	*p* = 0.006*	*d* = 0.372	1.8 ± 1.2		1.6 ± 1.1	*p* = 0.107	*d* = 0.244
AUDIT-C score	5.1 ± 2.5	5.3 ± 2.2	*p* = 0.648	*d* = 0.150	5.1 ± 2.6		5.3 ± 2.2	*p* = 0.676	*d* = 0.278
*Feelings of wellbeing and concern*									
Overall negative feelings score	9.7 ± 6.4	7.5 ± 6.0	*p* = 0.008*	*d* = 0.356	11.0 ± 7.0		7.6 ± 5.8	*p* < 0.001*	*d* = 0.556
Overall positive feelings score	9.2 ± 4.0	10.3 ± 3.8	*p* = 0.040*	*d* = 0.275	8.9 ± 4.3		10.2 ± 3.7	*p* = 0.035*	*d* = 0.321
*Irritability and anger*									
Anger/Out score	6.1 ± 3.5	6.0 ± 3.6	*p* = 0.791	*d* = 0.035	6.4 ± 3.6		5.9 ± 3.6	*p* = 0.390	*d* = 0.130
Anger/In score	7.4 ± 4.3	6.6 ± 3.5	*p* = 0.111	*d* = 0.213	8.0 ± 4.5		6.6 ± 3.6	*p* = 0.016*	*d* = 0.366
Overall anger score	13.5 ± 6.5	12.6 ± 5.3	*p* = 0.243	*d* = 0.161	14.4 ± 0.7		12.5 ± 5.5	*p* = 0.035*	*d* = 0.321

*SD* standard deviation, *AUDIT-C* Alcohol Use Disorders Identification Test

There was no association between years in sport and number of concussions in the rugby athletes (*r* = 0.028, *p* = 0.709). There was also no significant difference in years in sport between rugby athletes with or without at least three or five sport-related concussions (three: *p* = 0.992, five: *p* = 0.412). There were no significant differences in any of the mental health or wellbeing outcomes between those who had played rugby for < 25 versus ≥ 25 years (50th percentile) or < 30 versus ≥ 30 years (75th percentile; Table 9). There were also no differences when the 25th percentile cut-off was used (19 years).

**Table 9:** Mental health and wellbeing scores for retired rugby code players who played for more or less than 25 or 30 years (mean ± SD or percentage)

	At least 25 years of rugby (* n* = 90)		Difference	Effect size (Cohen’s *d*)	At least 30 years of rugby (* n* = 52)		Difference	Effect size (Cohen’s *d*)
	Yes	No			Yes	No		
*Mental health conditions*								
Irritability	51%	44%	*p* = 0.435		50%	47%	*p* = 0.701	
Depression	39%	44%	*p* = 0.534		39%	43%	*p* = 0.628	
Anxiety	32%	30%	*p* = 0.785		33%	31%	*p* = 0.858	
*Sleep*								
Difficulty falling asleep	1.0 ± 1.2	1.2 ± 1.1	*p* = 0.339	*d* = 0.148	1.0 ± 1.1	1.1 ± 1.1	*p* = 0.820	*d* = 0.039
Waking in the night and taking a long time to get back to sleep	1.6 ± 1.2	1.5 ± 1.2	*p* = 0.740	*d* = 0.050	1.7 ± 1.1	1.5 ± 1.3	*p* = 0.497	*d* = 0.106
Waking up too early	1.8 ± 1.1	1.6 ± 1.1	*p* = 0.183	*d* = 0.203	1.8 ± 1.0	1.7 ± 1.2	*p* = 0.325	*d* = 0.151
*Alcohol*								
AUDIT-C score	6.8 ± 4.4	7.1 ± 4.3	*p* = 0.768	*d* = 0.045	7.5 ± 4.1	6.8 ± 4.4	*p* = 0.345	*d* = 0.159
*Feelings of wellbeing and concern*								
Overall negative feelings score	8.8 ± 6.3	8.7 ± 6.8	*p* = 0.972	*d* = 0.005	8.2 ± 6.1	9.0 ± 6.8	*p* = 0.510	*d* = 0.111
Overall positive feelings score	9.6 ± 4.0	9.7 ± 3.9	*p* = 0.857	*d* = 0.027	10.0 ± 3.9	9.5 ± 3.9	*p* = 0.504	*d* = 0.112
*Irritability and anger*								
Anger/Out score	6.0 ± 3.4	6.1 ± 3.6	*p* = 0.816	*d* = 0.035	5.7 ± 2.9	6.2 ± 3.7	*p* = 0.366	*d* = 0.152
Anger/In score	6.9 ± 3.8	7.1 ± 3.9	*p* = 0.801	*d* = 0.038	6.2 ± 3.3	7.4 ± 4.0	*p* = 0.077	*d* = 0.298
Overall anger score	12.9 ± 6.0	13.2 ± 6.1	*p* = 0.766	*d* = 0.045	11.9 ± 5.0	13.5 ± 6.3	*p* = 0.096	*d* = 0.280

*SD* standard deviation, *AUDIT-C* Alcohol Use Disorders Identification Test

## Discussion

There is increasing concern surrounding the health and wellbeing of retired contact sports athletes, and the potential long-term effects of concussions and sub-concussive impacts on the brain. The notable findings reported in this study primarily indicated that the elite rugby code players experienced more adverse mental health outcomes in retirement and that there was an association with the number of prior concussions. Retired elite players also reported lower help-seeking behaviour.

Psychological signs of depression and anxiety were more prevalent in elite rugby code players. Overall, negative scores were higher and positive scores were lower in this group. It is noteworthy that one in five elite rugby code players reported that they would not turn to anyone if they had a problem or were upset about anything, with almost half suffering from, or having a history of, depression. In addition, one in ten elite rugby players reported that they often feel that “*life is hardly worth living*”*.* One possible contributing explanation for the greater prevalence of adverse mental health in former elite players, is injury-forced retirement. Elsewhere, retirement from an elite sports career, particularly if involuntary, has been associated with increased depressive symptoms [35–37]. In the current study, depression and anxiety were more prevalent in rugby players who retired because of injury when compared to those who did not retire due to injury. The psychosocial consequences of injury have been described elsewhere with former rugby players reporting a range of experienced emotions including shock, anger, financial worry, anxiety and a loss of identity [35].

Another possible contributing factor to the higher prevalence of adverse mental health in elite rugby players is repeated exposure to sub-concussive and concussive head impacts, given accumulating evidence associating repeated concussion and sub-concussive head impacts with poor brain outcomes and increased risk of neurodegenerative disease [38, 39]. Possible indicators of greater exposure to sub-concussive impacts are (1) participation in rugby at elite level, given the greater intensity of play and peak impact demands of 4.5–5.5 impacts min^−1^ [40–42] and/or (2) longer playing careers. While elite level rugby was associated with more adverse mental health outcomes following retirement, a longer playing career, at least over 19 years, was not. It is possible that a lower threshold of exposure to sub-concussive impacts exists, but it was not possible to explore this in the current study given that most players had engaged in a playing career that spanned ~ 25 years.

Former elite players reported significantly more concussions than amateur players, and an increasing number of concussions during a career was associated with more adverse mental health. Around half of former players who reported a history of five or more concussions reported suffering from current or previous depression or anxiety. For irritability, the prevalence was greater, with two-thirds of former players with five or more prior concussions reporting that they currently or previously suffered from irritability, double the prevalence of those reporting less than five concussions. The latter finding is further supported by observations of notably higher irritability and anger scores in those with five or more concussions. In studies of retired American football players, multiple concussions have also been associated with adverse psychological symptoms [17, 43]. In the general population, a recent large-scale meta-analysis found that mood disorders were around three times more prevalent in those who had experienced concussion, with symptoms persisting over decades [44]. Although in the current study causality could not be inferred, the pathophysiology linking mild traumatic brain injury to adverse psychological health has been suggested to involve neuroimmune alterations and cytokine dysregulation [45], reduced activation in the dorsolateral prefrontal cortex, and deactivation in the medial frontal and temporal regions [46]. Research has also demonstrated white matter abnormalities associated with depression in retired NFL players with a history of concussion [47]. The neurobiological linkage between concussion and long-term adverse behavioural symptoms requires further investigation.

Insufficient sleep, especially rapid eye movement-phase sleep, limits the brain’s processing of emotional information and can have serious consequences for psychological wellbeing and emotional reactivity (irritability, confusion, depression), including the risk of suicidal ideas or behaviours [48]. In the current study, sleep disruption was more prevalent in retired elite rugby players, with one in three reporting that they often, or always, woke too early, or had night awakenings and were unable to return to sleep. These disruptions were also more common in those who reported a history of three or more concussions. Sleep disruption, particularly frequent night awakenings, is common following concussion [49, 50]. In athletes and non-athletes, a higher number of prior concussions increases the likelihood of developing insomnia [52] and more severe insomnia scores [51], with symptoms persisting for months or years [49, 53]. Damage to the brain, increased intracranial pressure, and altered vascular tone following concussion contribute to sleep disruption [54]. Sleep disruption facilitates accumulation of hyperphosphorylated tau and amyloid plaques and reduces the ability of the glymphatic system to clear metabolic waste from the brain [55–57]. As a result, this may be implicated in the development of several chronic neurodegenerative diseases such as Alzheimer's disease and chronic traumatic encephalopathy. This association is bi-directional, and the accumulation of tau in the brain can impact sleep quality [57]. Further, addressing sleep disturbance may help reduce the risk of probable Alzheimer's disease [58].

In addition to sleep disruption, excessive alcohol consumption can exacerbate psychological distress and depression [59]. However, few studies have reported on the use of alcohol in former rugby players. Gouttebarge et al. [14] reported that 25% of former professional rugby union players reported adverse alcohol use. In current rugby league players, 68% reported hazardous use of alcohol [12] and in current professional rugby union players, those who sustained two or more concussions during one season were more likely to engage in adverse alcohol use and experience symptoms of depression [11]. In contrast, we found no differences between groups for the AUDIT-C score, although former elite rugby code players reported drinking alcohol less frequently than non-contact athletes. However, more than one in two former athletes, regardless of sport, had AUDIT-C scores that indicated a higher risk from alcohol consumption.

This study helps to address the lack of evidence on the prevalence of adverse psychological health in retired rugby code players and in relation to history of concussion. There are several potential limitations to note. First, as with similar studies involving the recruitment of volunteers, the study was subject to non-response or selection bias. Therefore, results may not be entirely generalisable to all rugby players, although the inclusion of both elite and amateur-level former rugby code players increases the applicability of the study findings. Second, the cross-sectional design did not enable us to make causal inferences, and longitudinal study designs that provide higher-quality evidence of relationships between variables are warranted. However, there are experimental and imaging studies that have demonstrated associations between concussion, structural and functional brain alterations, sleep disruption and psychological symptomology [46, 47, 51, 54]. Third, it should be considered that while the questions were derived from validated clinical tools, individual items from these tools have not been separately validated. We included statistical analysis of individual items in addition to overall score because each provided distinct and insightful information for the current study. Fourth, although an unavoidable limitation of this research was retrospective reporting of concussions, moderate reliability has been demonstrated for the retrospective reporting of concussions by NFL players over a time gap of 9 years [60].

## Conclusions

Psychological signs of anxiety and depression and difficulties with sleep were consistently more prevalent in former elite rugby code players and were associated with a higher number of concussion injuries. Former athletes with a history of concussion had higher negative feeling scores (≥ 3 concussions), irritability and anger (≥ 5 concussions). These outcomes are likely interrelated and future research should now advance knowledge on the behavioural effects of mild traumatic brain injury to understand the experience of mental health in rugby, factoring in psychosocial aspects of retirement and identification of possible neurobiological and neurochemical factors. In practical terms, our findings suggest that timely strategies are needed to address mental health and sleep disturbances in retired elite rugby code players, who are also less likely to seek help should they need it.

## Supplementary Information

Below is the link to the electronic supplementary material.

Supplementary file1 (DOCX 29 KB)

## Declarations

### Data availability

The general health questionnaire and the datasets generated during the current study are not publicly available but are available from the corresponding author on reasonable request.

### Code availability

Data are not publicly available. Code used for study analyses may be made available upon reasonable request to the corresponding author.
