# Supplementary material for: Mental Health and Wellbeing of Retired Elite and Amateur Rugby Players and Non-contact Athletes and Associations with Sports-Related Concussion: The UK Rugby Health Project
Source: Sports Med. 2021 Nov 18;52(6):1419–31. doi: 10.1007/s40279-021-01594-8 (PMC9124647; doi:10.1007/s40279-021-01594-8)
Supplement: Supplementary file 1 — Supplementary file1 (DOCX 29 KB) [file 40279_2021_1594_MOESM1_ESM.docx]

**Sports Medicine**

**Supplementary Information:**

**Mental health and wellbeing of retired elite and amateur rugby players and non-contact athletes and associations with sports-related concussion: the UK Rugby Health Project**

Karen Hind^*^, Natalie Konerth, Ian Entwistle, Patria Hume, Alice Theadom, Gwyn Lewis, Doug King, Thomas Goodbourn, Marianna Bottiglieri, Paula Ferraces-Riegas, Amanda Ellison, Paul Chazot.

Corresponding author: Dr K. Hind, Department of Sport and Exercise Sciences, Durham University, United Kingdom. Email: [karen.hind@durham.ac.uk](mailto:karen.hind@durham.ac.uk).

Tables A1a and A1b provide the full data for individual sub-scale items.

*A1a. Irritability and anger for participants in elite rugby codes, amateur rugby codes and*

*non-contact sport codes (mean ± standard deviation)*

|  | **Elite rugby codes** | **Amateur rugby codes** | **Non-contact athletes** | **Difference** |
| --- | --- | --- | --- | --- |
| I express my anger | 1.2 ± 0.9 | 1.1 ± 0.9 | 1.1 ± 1.0 | p=0.686 |
| I keep things in | 1.6 ± 0.9 | 1.5 ± 0.9 | 1.7 ± 0.9 | p=0.470 |
| I sulk | 1.0 ± 0.8 | 0.6 ± 0.7 | 0.9 ± 0.7 | ER v AR p=0.002  ER v NC p=0.539  AR v NC p=0.113 |
| I withdraw from people | 1.1 ± 0.9 | 0.8 ± 0.8 | 0.9 ± 0.7 | ER v AR p=0.031  ER v NC p=0.405  AR v NC p=0.587 |
| I make sarcastic remarks to others | 1.4 ± 0.9 | 1.3 ± 0.8 | 1.1 ± 0.9 | p=0.214 |
| I do things like slam doors | 0.5 ± 0.8 | 0.5 ± 0.6 | 0.4 ± 0.6 | p=0.659 |
| I boil inside but don't show it | 1.0 ± 0.9 | 0.9 ± 0.9 | 1.1 ± 0.9 | p=0.326 |
| I argue with others | 0.9 ± 0.7 | 0.9 ± 0.7 | 0.9 ± 0.6 | p=0.978 |
| I tend to harbour grudges that I don't tell anyone about | 0.7 ± 0.9 | 0.6 ± 0.8 | 0.6 ± 0.8 | p=0.517 |
| I strike out at whatever infuriates me | 0.3 ± 0.6 | 0.2 ± 0.5 | 0.3 ± 0.6 | p=0.788 |
| I am secretly quite critical of others | 0.9 ± 0.9 | 0.8 ± 0.7 | 0.9 ± 0.7 | p=0.717 |
| I am angrier than I am willing to admit | 1.0 ± 1.0 | 0.9 ± 0.8 | 0.8 ± 0.9 | p=0.666 |
| I say nasty things | 0.6 ± 0.7 | 0.5 ± 0.6 | 0.4 ± 0.6 | p=0.508 |
| I'm irritated a great deal more than people are aware of | 1.1 ± 1.0 | 1.0 ± 0.8 | 0.9 ± 0.8 | p=0.523 |
| I lose my temper | 0.7 ± 0.8 | 0.7 ± 0.7 | 0.5 ± 0.6 | p=0.266 |
| **Anger/Out score** | 6.5 ± 3.8 | 5.8 ± 3.5 | 5.5 ± 3.3 | p=0.254 |
| **Anger/In score** | 7.4 ± 4.3 | 6.5 ± 3.6 | 7.1 ± 3.6 | p=0.255 |
| **Overall anger score** | 13.8 ± 6.7 | 12.3 ± 5.6 | 12.6 ± 4.6 | p=0.172 |

*Responses were coded as follows: 0-Never, 1-Sometimes, 2-Often, 3-Always; SD = Standard Deviation*

*A1b. Mental health and wellbeing scores for former athletes with or without at least 3 or 5 sports-related concussion (mean±SD or %)*

|  | At least 3 sport-related concussions (n=99) | | Difference | At least 5 sport-related concussions (n=60) | | Difference |
| --- | --- | --- | --- | --- | --- | --- |
|  | Yes | No |  | Yes | No |  |
| **Mental Health Conditions** | | | | | | |
| Irritability | 54% | 30% | p=0.001 | 63% | 33% | p<0.001 |
| Depression | 47% | 30% | p=0.010 | 55% | 30% | p=0.001 |
| Anxiety | 37% | 27% | p=0.116 | 44% | 26% | p=0.012 |
| **Sleep** | | | | | | |
| Difficulty falling asleep | 1.2 ± 1.2 | 1.0 ± 1.0 | p=0.157 | 1.3 ± 1.3 | 1.0 ± 1.0 | p=0.133 |
| Waking in the night and taking a long time to get back to sleep | 1.8 ± 1.1 | 1.2 ± 1.2 | p<0.001 | 1.8 ± 1.2 | 1.4 ± 1.2 | p=0.030 |
| Waking up too early | 1.9 ± 1.1 | 1.5 ± 1.1 | p=0.006 | 1.8 ± 1.2 | 1.6 ± 1.1 | p=0.107 |
| **Alcohol Consumption** | | | | | | |
| How often do you drink? | 2.4 ± 1.2 | 2.6 ± 1.0 | p=0.226 | 2.3 ± 1.2 | 2.6 ± 1.0 | p=0.104 |
| How many drinks do you have on a typical day when drinking? | 4.0 ± 2.5 | 3.9 ± 2.2 | p=0.838 | 4.2 ± 2.6 | 3.9 ± 2.3 | p=0.447 |
| How often do you have 6 or more drinks on one occasion? | 1.5 ± 1.0 | 1.4 ± 1.0 | p=0.601 | 1.5 ± 1.0 | 1.5 ± 1.0 | p=0.888 |
| How often during the last 12 months have you found you were unable to stop drinking once you started? | 0.4 ± 0.9 | 0.2 ± 0.6 | p=0.056 | 0.5 ± 1.0 | 0.2 ± 0.6 | p=0.021 |
| How often during the last 12 months have you been unable to remember what happened the night before because you had been drinking? | 0.5 ± 0.8 | 0.4 ± 0.6 | p=0.142 | 0.6 ± 0.9 | 0.4 ± 0.6 | p=0.079 |
| Overall Alcohol Score | 7.3 ± 5.0 | 6.6 ± 3.5 | p=0.290 | 7.7 ± 5.7 | 6.6 ± 3.5 | p=0.147 |
| **Feelings of Wellbeing and Concern** |  |  |  |  |  |  |
| Everything is going right for me | 1.3 ± 0.8 | 1.4 ± 0.7 | p=0.106 | 1.3 ± 0.8 | 1.4 ± 0.7 | p=0.249 |
| I’ve made a mess of things again | 0.7 ± 0.7 | 0.6 ± 0.7 | p=0.112 | 0.8 ± 0.8 | 0.6 ± 0.7 | p=0.007 |
| Satisfied | 1.5 ± 0.8 | 1.7 ± 0.7 | p=0.088 | 1.5 ± 0.8 | 1.7 ± 0.7 | p=0.060 |
| Miserable | 1.0 ± 0.7 | 0.7 ± 0.7 | p=0.006 | 1.1 ± 0.8 | 0.7 ± 0.7 | p=0.002 |
| My life is on the right track | 1.5 ± 0.9 | 1.7 ± 0.8 | p=0.110 | 1.5 ± 0.9 | 1.7 ± 0.8 | p=0.155 |
| Nothing is much fun anymore | 0.8 ± 0.8 | 0.5 ± 0.7 | p=0.005 | 1.0 ± 0.9 | 0.6 ± 0.7 | p=0.001 |
| Confident | 1.6 ± 0.8 | 1.8 ± 0.7 | p=0.034 | 1.6 ± 0.9 | 1.8 ± 0.7 | p=0.104 |
| Depressed | 0.6 ± 0.8 | 0.5 ± 0.7 | p=0.146 | 0.8 ± 0.9 | 0.5 ± 0.7 | p=0.044 |
| Nothing goes right with me | 0.6 ± 0.7 | 0.4 ± 0.7 | p=0.114 | 0.7 ± 0.8 | 0.4 ± 0.7 | p=0.024 |
| Happy | 1.7 ± 0.7 | 1.8 ± 0.7 | p=0.098 | 1.6 ± 0.7 | 1.8 ± 0.7 | p=0.102 |
| Down | 0.9 ± 0.6 | 0.8 ± 0.7 | p=0.077 | 1.1 ± 0.6 | 0.8 ± 0.6 | p=0.002 |
| Life is hardly worth living | 0.4 ± 0.6 | 0.2 ± 0.5 | p=0.060 | 0.5 ± 0.7 | 0.2 ± 0.5 | p=0.006 |
| Content | 1.7 ± 0.8 | 1.9 ± 0.8 | p=0.121 | 1.6 ± 0.8 | 1.8 ± 0.8 | p=0.028 |
| Upset | 0.9 ± 0.7 | 0.8 ± 0.6 | p=0.267 | 1.0 ± 0.7 | 0.8 ± 0.6 | p=0.034 |
| Lost sleep over worry | 1.0 ± 0.8 | 0.7 ± 0.8 | p=0.006 | 1.0 ± 0.9 | 0.7 ± 0.8 | p=0.033 |
| Unable to concentrate | 0.9 ± 0.7 | 0.7 ± 0.7 | p=0.018 | 1.1 ± 0.7 | 0.7 ± 0.7 | p=0.001 |
| Unable to make decisions | 0.8 ± 0.7 | 0.5 ± 0.7 | p=0.006 | 1.0 ± 0.7 | 0.5 ± 0.7 | p<0.001 |
| Pressured | 1.1 ± 0.7 | 1.0 ± 0.8 | p=0.168 | 1.1 ± 0.7 | 1.0 ± 0.8 | p=0.398 |
| Overall negative feelings score | 9.7 ± 6.4 | 7.5 ± 6.0 | p=0.008 | 11.0 ± 7.0 | 7.6 ± 5.8 | p=0.001 |
| Overall positive feelings score | 9.2 ± 4.0 | 10.3 ± 3.8 | p=0.040 | 8.9 ± 4.3 | 10.2 ± 3.7 | p=0.035 |
| **Irritability and Anger** |  |  |  |  |  |  |
| I express my anger | 1.2 ± 0.9 | 1.1 ± 0.9 | p=0.455 | 1.1 ± 1.0 | 1.1 ± 0.9 | p=0.882 |
| I keep things in | 1.6 ± 0.9 | 1.5 ± 0.9 | p=0.389 | 1.7 ± 1.0 | 1.5 ± 0.8 | p=0.393 |
| I sulk | 0.8 ± 0.8 | 0.8 ±0.7 | p=0.896 | 1.0 ± 0.8 | 0.8 ± 0.7 | p=0.056 |
| I withdraw from people | 1.1 ± 0.9 | 0.9 ± 0.8 | p=0.081 | 1.3 ± 0.9 | 0.8 ± 0.8 | p<0.001 |
| I make sarcastic remarks to others | 1.3 ± 0.8 | 1.3 ± 0.9 | p=0.535 | 1.4 ± 0.7 | 1.3 ± 0.8 | p=0.412 |
| I do things like slam doors | 0.4 ± 0.7 | 0.4 ± 0.7 | p=0.845 | 0.4 ± 0.7 | 0.4 ± 0.7 | p=0.919 |
| I boil inside but don't show it | 1.0 ± 0.9 | 0.9 ± 0.9 | p=0.350 | 1.1 ± 0.9 | 0.9 ± 0.9 | p=0.314 |
| I argue with others | 0.9 ± 0.7 | 0.9 ± 0.7 | p=0.449 | 0.9 ± 0.7 | 0.9 ± 0.7 | p=0.649 |
| I tend to harbour grudges that I don't tell anyone about | 0.6 ± 0.8 | 0.7 ± 0.8 | p=0.852 | 0.7 ± 0.8 | 0.6 ± 0.8 | p=0.766 |
| I strike out at whatever infuriates me | 0.2 ± 0.5 | 0.3 ± 0.5 | p=0.950 | 0.2 ± 0.4 | 0.3 ± 0.6 | p=0.231 |
| I am secretly quite critical of others | 1.0 ± 0.8 | 0.8 ± 0.7 | p=0.066 | 1.1 ± 0.8 | 0.8 ± 0.8 | p=0.042 |
| I am angrier than I am willing to admit | 0.9 ± 1.0 | 0.9 ± 0.9 | p=0.837 | 1.0 ± 1.0 | 0.9 ± 0.9 | p=0.494 |
| I say nasty things | 0.5 ± 0.7 | 0.5 ± 0.6 | p=0.492 | 0.6 ± 0.8 | 0.5 ± 0.6 | p=0.352 |
| I'm irritated a great deal more than people are aware of | 1.1 ± 1.0 | 0.9 ± 0.8 | p=0.061 | 1.2 ± 1.0 | 0.9 ± 0.8 | p=0.055 |
| I lose my temper | 0.7 ± 0.7 | 0.6 ± 0.7 | p=0.549 | 0.7 ± 0.8 | 0.6 ± 0.7 | p=0.446 |
| Anger out score | 6.1 ± 3.5 | 6.0 ± 3.6 | p=0.791 | 6.4 ± 3.6 | 5.9 ± 3.6 | p=0.390 |
| Anger in score | 7.4 ± 4.3 | 6.6 ± 3.5 | p=0.111 | 8.0 ± 4.5 | 6.6 ± 3.6 | p=0.016 |
| Overall anger score | 13.5 ± 6.5 | 12.6 ± 5.3 | p=0.242 | 14.4 ± 6.7 | 12.5 ± 5.5 | p=0.035 |
| **Attachments** |  |  |  |  |  |  |
| Involvement with family | 3.9 ± 1.3 | 4.2 ± 1.0 | p=0.111 | 3.8 ± 1.4 | 4.2 ± 1.1 | p=0.103 |
| Involvement with friends | 3.6 ± 1.3 | 3.6 ± 1.2 | p=0.673 | 3.6 ± 1.3 | 3.6 ± 1.2 | p=0.816 |
| Involvement with work/university | 3.2 ± 1.7 | 3.3 ± 1.5 | p=0.854 | 3.2 ± 1.8 | 3.3 ± 1.5 | p=0.620 |
| Involvement with former sports friends/clubs | 2.2 ± 1.7 | 2.2 ± 1.7 | p=0.920 | 1.9 ± 1.6 | 2.3 ± 1.7 | p=0.149 |
